# Penetrative trace fossils from the late Ediacaran of Mongolia: early onset of the agronomic revolution

**DOI:** 10.1098/rsos.172250

**Published:** 2018-02-28

**Authors:** Tatsuo Oji, Stephen Q. Dornbos, Keigo Yada, Hitoshi Hasegawa, Sersmaa Gonchigdorj, Takafumi Mochizuki, Hideko Takayanagi, Yasufumi Iryu

**Affiliations:** 1 Nagoya University Museum, Nagoya University, Nagoya 464-8601, Japan; 2 Department of Geosciences, University of Wisconsin-Milwaukee, Milwaukee, WI 53211, USA; 3 Graduate School of Environmental Studies, Nagoya University, Nagoya 464-8601, Japan; 4 Faculty of Science and Technology, Kochi University, Kochi 780-8520, Japan; 5 Mongolian University of Science and Technology, Ulaanbaatar 46/520, Mongolia; 6 Iwate Prefectural Museum, Morioka 020-0102, Japan; 7 Department of Earth Science, Tohoku University, Sendai 980-8578, Japan

**Keywords:** Bayan Gol, Zuun-arts formation, *Arenicolites*, Cambrian explosion, Cambrian agronomic revolution, bilaterians

## Abstract

The Cambrian radiation of complex animals includes a dramatic increase in the depth and intensity of bioturbation in seafloor sediment known as the ‘agronomic revolution’. This bioturbation transition was coupled with a shift in dominant trace fossil style from horizontal surficial traces in the late Precambrian to vertically penetrative trace fossils in the Cambrian. Here we show the existence of the first vertically penetrative trace fossils from the latest Ediacaran: dense occurrences of the U-shaped trace fossil *Arenicolites* from late Precambrian marine carbonates of Western Mongolia. Their Ediacaran age is established through stable carbon isotope chemostratigraphy and their occurrence stratigraphically below the first appearance of the trace fossil *Treptichnus pedum*. These *Arenicolites* are large in diameter, penetrate down to at least 4 cm into the sediment, and were presumably formed by the activity of bilaterian animals. They are preserved commonly as paired circular openings on bedding planes with maximum diameters ranging up to almost 1 cm, and as U- and J-shaped tubes in vertical sections of beds. Discovery of these complex penetrative trace fossils demonstrates that the agronomic revolution started earlier than previously considered.

## 1. Introduction

The Precambrian–Cambrian transition is one of the most dramatic periods in the history of life because of the rapid diversification of complex animal life, often called the ‘Cambrian Explosion.’ This transition included profound changes in animal behaviour, including increased depth and intensity of burrowing in the seafloor [1]. This increase in bioturbation created important changes in the nature of marine substrates. In the final interval of the Precambrian, the Ediacaran Period, benthic animals were adapted to living on, or under firm, microbial-mat-bound seafloors. In the Cambrian, the infaunal activity of animals accelerated and substrates gradually became more mixed, producing soft substrates with high water and oxygen content, as well as a surface mixed layer [2]. This dramatic change is known as the ‘agronomic revolution’ [1].

Alongside the general increase in bioturbation levels, the dominant style of trace fossil also changed from the Ediacaran to the Cambrian. Ediacaran trace fossils are mostly horizontal (parallel to bedding), and there are no vertical trace fossils that penetrate the sediment [3]. They are also generally small and their bilaterian origin has been questioned [4]. Penetrative trace fossils become common in the Cambrian with the first stratigraphic occurrence of the penetrative trace fossil *Treptichnus pedum* defining the Precambrian–Cambrian boundary [5], which has a relatively wide environmental tolerance [6]. This change in the style of trace fossils is regarded as reflecting an important evolutionary innovation in animal behaviour, with increased morphological diversity, expanded size range and increased sediment penetration in the Cambrian [7]. More diverse and deeply penetrative trace fossils become dominant in Cambrian Stage 2 [8,9]. Here we report from Western Mongolia the first reliable occurrence of abundant penetrative trace fossils from the latest Ediacaran, indicating that this evolutionary innovation took place earlier than previously thought, and providing trace fossil evidence for Precambrian bilaterians with complex behavioural patterns.

## 2. Geologic setting

**Figure 1.**
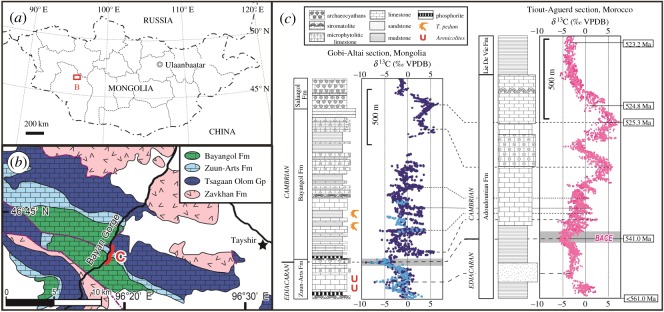
Locality information and stable isotope chemostratigraphy. (*a*) Locality within Mongolia. (*b*) Locality and geologic map of Bayan Gol Valley in Gobi-Altai Province, modified from Smith *et al*. [10]. Bayan Gol Fm: Siliciclastics and carbonates, early Cambrian; Zuun-Arts Fm: Carbonates with minor siliciclastics, Ediacaran; Tsagaan Olom Gp: Diamictites and carbonates, Cryogenian-Ediacaran; Zavkhan Fm: Felsic volcanics, Cryogenian. (*c*). Carbon isotope (*δ*^13^C) curves of the Zuun-Arts Formation and the lower part of the Bayangol Formation at Bayan Gol Valley, with a possible correlation with the Moroccan section [11]. Note first occurrence of *Treptichnus pedum* and the Precambrian–Cambrian boundary negative excursion are both above the horizons of *Arenicolites*. Blue dots by our isotope measurements, and dark blue ones from a composite carbon isotopic curve from Zavkhan terrane by Smith *et al*. [10].

**Figure 2.**
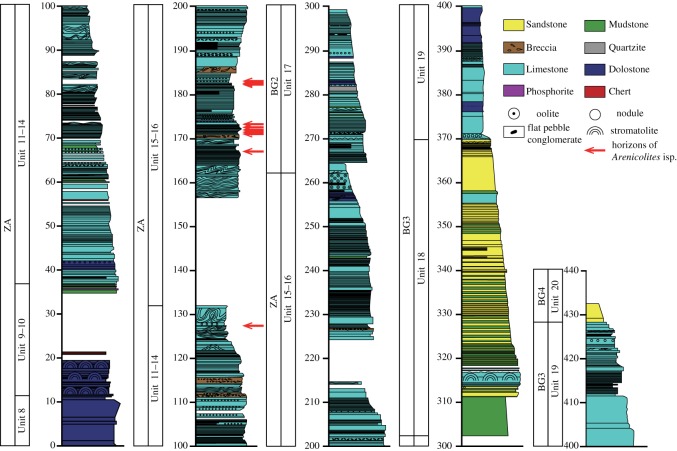
Geologic column of the upper part of the Zuun-Arts Formation (Units 8–17), with the horizons of *Arenicolites* (red arrows). Note that slump beds and flat-pebble conglomerates are common near the *Arenicolites* horizons. Division of Units 8–20 by [13] and division ZA, BG2-BG4 by [10].

Well-preserved and nearly continuous Ediacaran to Cambrian marine sedimentary rocks are exposed along Bayan Gol Valley, Govi-Altay, Western Mongolia (figure 1) [10,12]. Previous workers divided the beds of the Bayan Gol Valley section into two formations; Tsagaan Oloom and Bayan Gol formations in ascending order, and further subdivided them into numbered units [13] (figure 2). The base of the Bayan Gol Formation was placed at the base of the Unit 18 [12,13]. Initial trace fossil studies placed the likely Precambrian–Cambrian boundary near the base of the Bayan Gol Formation because of the first occurrence of the trace fossil *Treptichnus pedum* at this level (e.g. [14]). Recently, the stratigraphy of the upper part of the Tsagaan Oloom and the Bayan Gol formations was revised, and the position of the Precambrian–Cambrian boundary was refined as being in the uppermost part of the Zuun-Arts Formation (upper part of the former ‘Tsagaan Oloom Formation’) because of the existence there of a remarkable negative excursion with the minimum value less than −5‰ in the stable carbon isotope profile interpreted as the BACE boundary excursion [10,15,16], as well as the existence of relatively positive carbon isotope ratio values below the negative spike. The boundary of the two formations, i.e. the Zuun-Arts and the Bayangol (former ‘Bayan Gol’) formations, was recently revised and moved to the lower horizon [10,16]. A high-resolution stable carbon isotope profile with a 2 m interval produced in this study confirms the presence of the boundary excursion stratigraphically above the *Arenicolites* described here (figure 1). The integrated stable carbon isotope profile of the Gobi-Altai succession enables correlation with the stable carbon isotope profile of the well-dated boundary succession in Morocco. This regional correlation further confirms that the Precambrian–Cambrian boundary can conclusively be placed in the uppermost Zuun-Arts Formation [10,11] (figure 1).

## 3. Material and methods

Trace fossils were observed on 11 bedding plane surfaces, as well as in multiple vertical cross-sections, of bedded limestones of the upper part of the Zuun-Arts Formation in the middle portion of the Bayan Gol Valley section (figure 2). Several bedding plane samples were collected and returned to the laboratory. Isolated samples with well-preserved trace fossils were also collected from float. The diameters of the circular trace cross-sections were measured on six separate bedding planes to analyse their size distribution.

In order to confirm the three-dimensional structure of the burrows inside the samples, some blocks were serially dissected perpendicular to bedding with a rock saw at an interval of 7 mm (1 mm was lost between each slab during cutting). The surfaces were polished and scanned by digital scanner (Canon MG7500). Scanned images of all the surfaces were superimposed to reveal the position of burrows on each slab and then to clarify the entire shape of the burrows. This technique was employed because our attempt to use CT scanning failed to reveal the burrow structure, probably due to very minor difference of densities between the matrix and the material inside the burrows.

Micrograph observations of vertical cross-sections and horizontal cross-sections were made from the sample collected at 181.36 m above the base of Unit 8. These thin sections span the boundary of the burrow and the matrix in order to determine if the boundary is clear and unlined, or if there is overgrowth of lining cements.

Samples from the trace-bearing carbonates were analysed with a Rigaku Multiflex X-ray diffractometer (XRD) at the Nagoya University Museum, Japan to determine mineral abundances and assess dolomitization levels. Additional XRD analyses were made for the sample from 181.36 m, for the black limestone matrix and light-coloured material inside the burrow.

In order to confirm the stratigraphic location of the Precambrian–Cambrian boundary for this study, a stable C isotope (*δ*^13^C) curve was created at a 2 m sample interval through the carbonate facies of Units 8–16 of the Zuun-Arts Formation and Units 17–19 of the Bayangol Formation in Bayan Gol Valley (figure 1*c*). Carbon isotopic ratios were measured with a ThermoFisher DeltaV Advantage mass spectrometer, coupled with a ThermoQuest Kiel-III automated carbonate device, at the Department of Earth Science, Tohoku University. The external precision determined by replicate analysis of the laboratory standard was 0.02‰ for *δ*^13^C values (1*σ*).

## 4. Results

**Figure 3.**
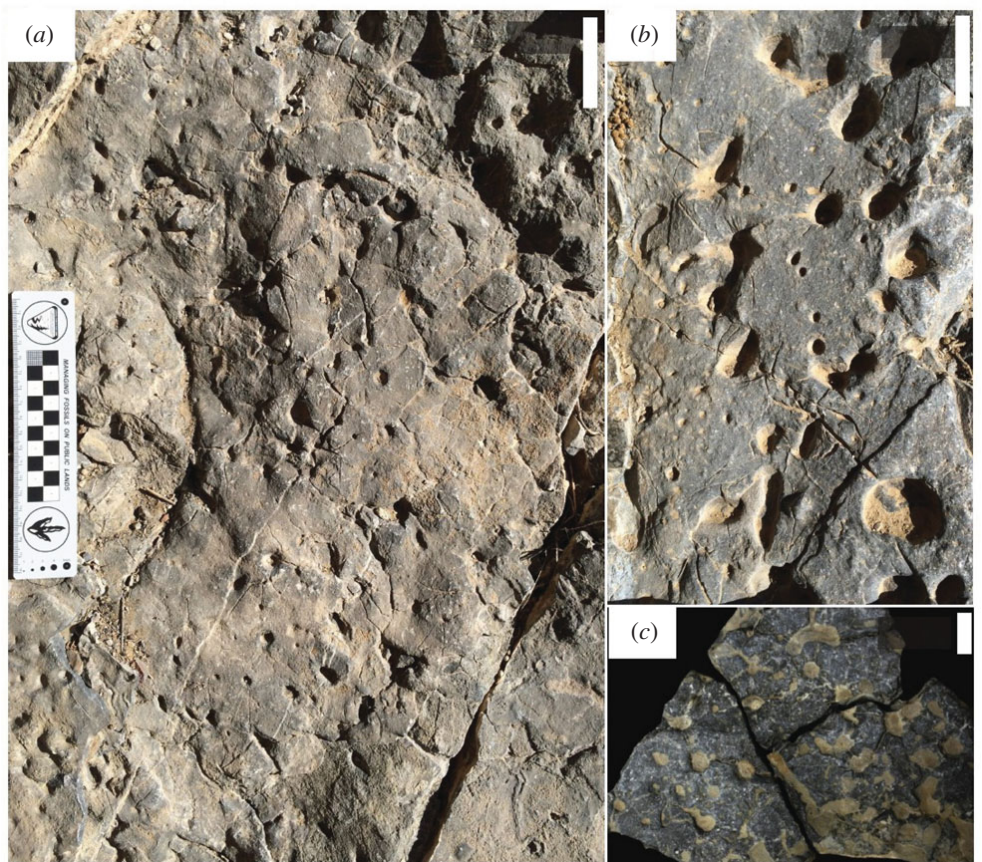
Bedding plane views of *Arenicolites* trace fossils from Unit 15 of the Zuun-Arts Formation, Bayan Gol Valley. (*a*) Bedding plane at 166.68 m above base of Unit 8. Note dense circular openings. Scale bar is 5 cm. (*b*) Part of bedding plane at 182.88 m above the base of Unit 8 showing abundant circular, paired openings. Also note arcuate impressions from bases of U-shaped *Arenicolites* from eroded beds above. Scale bar is 5 cm. (*c*) Part of bedding plane at 181.36 m above the base of Unit 8 showing paired circular openings infilled with sediment as well as arcuate traces. Note one arcuate burrow on the upper part of this photo, suggesting the base of an *Arenicolites* burrow. Scale bar is 2 cm.

**Figure 4.**
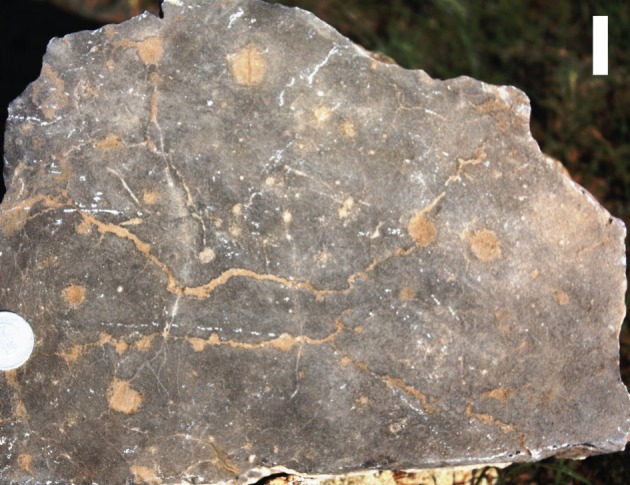
Section of bedding plane from float adjacent to outcrop containing paired circular trace fossils identifiable as *Arenicolites*. Scale bar is 2 cm.

**Figure 5.**
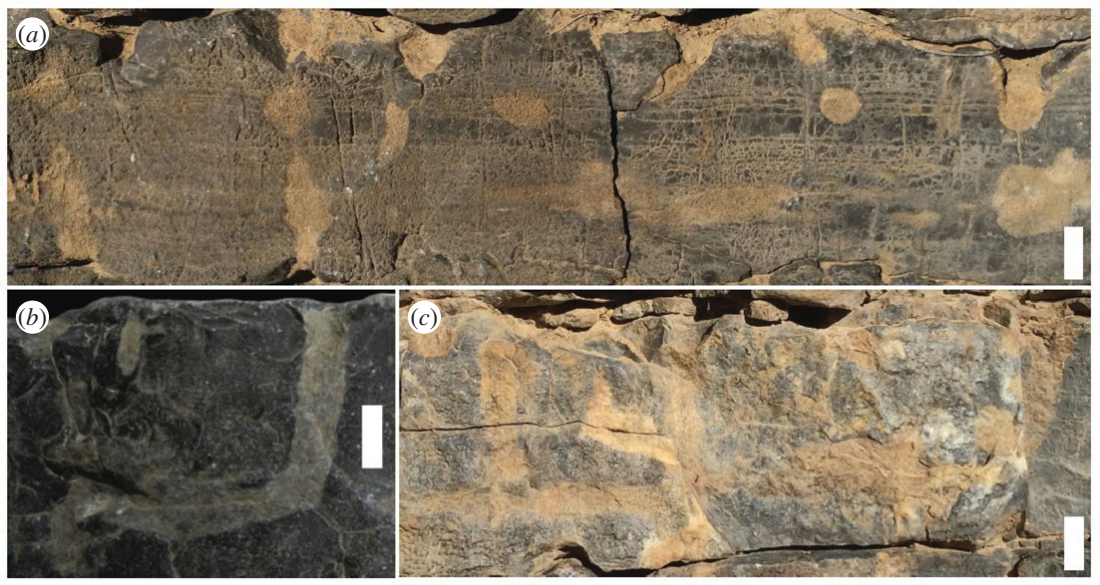
Vertical cross-section views of *Arenicolites* trace fossils from Unit 15 of the Zuun-Arts Formation, Bayan Gol Valley. All scale bars are 1 cm. (*a*) Side of bed showing ichnofabric of vertical to sub-vertical traces from vertical shafts of the U-shaped burrow and circular cross-sections of horizontal traces from base of U-shaped burrow. (*b*) Side of bed showing J-shaped cross-section of *Arenicolites*. Compression has made trace more angular. (*c*) Side of bed showing moderately dense ichnofabric (ii3) of vertical, sub-vertical and horizontal traces.

**Figure 6.**
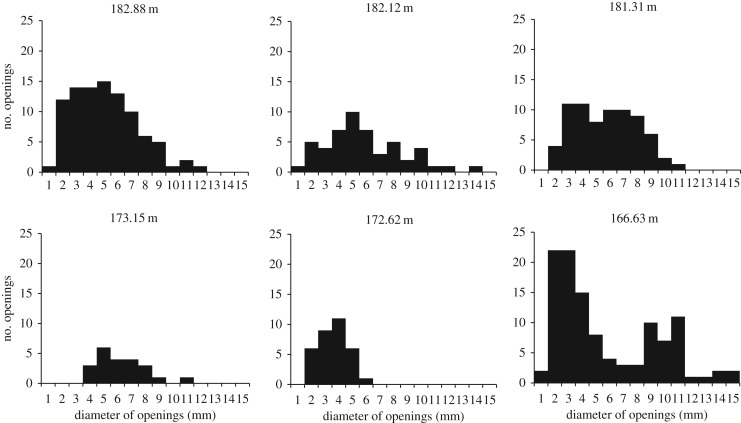
Size distribution of the circular opening of *Arenicolite*s trace fossils on bedding surfaces from six different horizons. Horizons are shown by the height above the base of the Unit 8.

Eleven beds of Units 14, 15 and 16 from the upper part of the Zuun-Arts Formation contain abundant trace fossils on the bedding planes (figures 2, 3 and 4), which are also visible on the sides (figure 5), and occasionally on the bottoms of beds. These traces are circular to sub-circular openings on bedding surfaces that range in diameter from less than 1 mm to greater than 1 cm (figure 3). There are two modes in the diameters of openings on one of the bedding planes, but there is no clear bimodal distribution on the other horizons examined in this way (figure 6). Many similar-sized openings appear to be paired, consistent with U-shaped burrows. These paired openings also occur in pieces of float adjacent to the outcrop (figure 4). Small concave arcuate traces occur on these bedding planes as well, and they resemble the bases of U-shaped burrows from overlying beds that have eroded away (figure 3*b,c*). The horizontal trace fossil *Planolites* is also preserved on these bedding planes.

**Figure 7.**
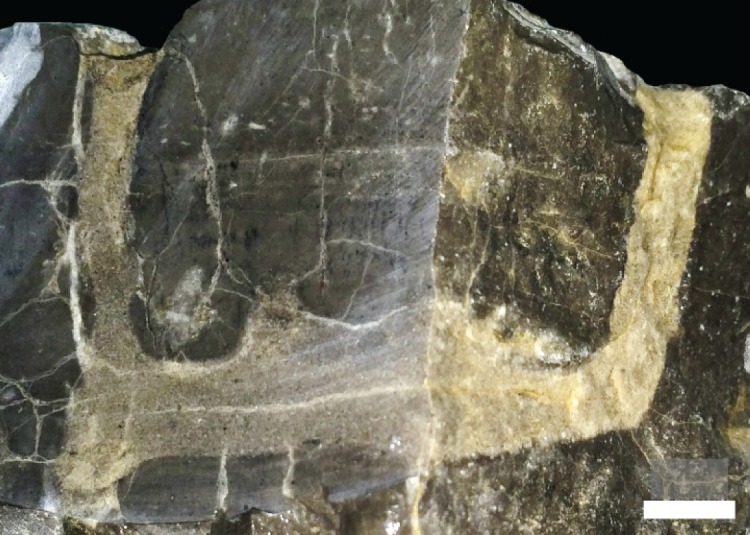
Full vertical U-shaped morphology of J-shaped trace fossil from figure 5*b* revealed through vertical sectioning with rock saw with two oblique cuts. Readily identifiable as *Arenicolites*. Scale bar = 1 cm.

Vertical exposures of some beds reveal that these circular openings are the tops of infilled vertical shafts penetrating the limestone, which sometimes form J-shaped traces (figure 5). These tubes are unlined and lack spreiten. Maximum depth of penetration of these tubes is about 4 cm. Considering compaction of the muddy limestone, originally they probably extended deeper into the sediment. The tubes are infilled with light-coloured sediment that differs from the matrix, indicating that the matrix was firm and the tubes were open at the time of formation. The traces are dense enough to form a moderately intense ichnofabric in vertical outcrop (ichnofabric index (ii) = 3 [17]). No full U-shaped burrows were observed in these vertical cross-sections in the field. Sectioning one conspicuous J-shaped trace in the lab with a rock saw revealed its full U-shaped morphology (figure 7).

**Figure 8.**
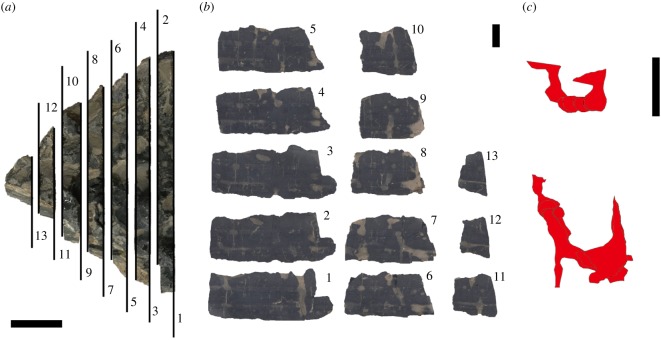
Two burrow morphologies reconstructed by superimposing 13 images from slabbed surfaces of a block. (*a*) Seven parallel slabs were cut with a rock saw, thickness of each slab being 7 mm and the lost interval between adjacent slabs being approximately 1 mm. (*b*) Scanned images of thirteen surfaces. Image of even numbers are horizontally inverted. (*c*) Burrow shapes (red) from the 13 superimposed images. Both burrows show clear U-shaped morphology, with an irregular burrow wall, probably due to later deformation. These burrow shapes are aligned horizontally but shown here vertically for convenience. All scale bars are 2 cm.

In order to further reconstruct the three-dimensional morphology of burrows in one sample, the block was cut perpendicular to bedding into seven parallel slabs. By stacking 13 scanned images of these vertical surfaces, internal morphologies of two burrows were observed (figure 8). These two burrows are U-shaped, with irregular boundaries. These burrows were probably originally more uniformly U-shaped, but presumably were subsequently deformed, indicating weak strength of the burrow walls. The vertical shafts are also observed penetrating the bases of several beds. Based on their morphological characteristics observed on bedding planes (paired circular openings and arcuate traces) and on their vertical cross-sectional morphology (U-shaped), these traces are interpreted as the penetrative burrow *Arenicolites*.

The limestones containing these *Arenicolites* show common evidence for synsedimentary soft-sediment deformation. Beds are folded and contorted, including some of the trace-bearing beds, indicating slumping (figure 2). Flat-pebble conglomerates are common in the units where *Arenicolites* burrows are abundant (figure 2). They are often interpreted as evidence of shallow marine deposition above storm wave base. However, the existence of large-scale, synsedimentarily deformed slump beds suggests a rather deep continental slope setting. One locality near the *Arenicolites* horizons shows a sudden lateral change of bed forms from non-disturbed, intact bedded limestone to a slump bed with brecciated limestone slabs in muddy matrix, suggesting that these slumps finally produced flat-pebble conglomerates from bedded limestones on the slope.

**Figure 9.**
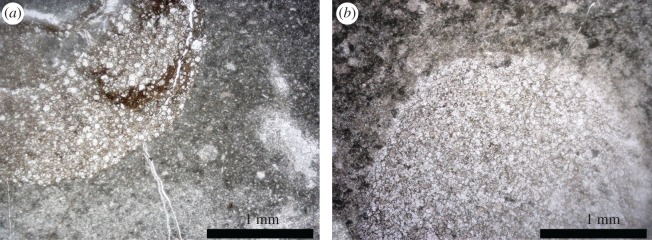
Thin section micrographs (plane polarized) of *Arenicolites* burrows. (*a*). Vertical view of a curved burrow, from vertical shaft (upper) to horizontal tunnel (left). (*b*). Horizontal view of a burrow with circular outline. Both micrographs show micritic matrix and relatively coarse-grained carbonates in burrows, with quartz grains of probable clastic origin. Both scale bars are 1 mm.

Micrographs at the boundary of *Arenicolites* burrow walls and the matrix, as well as XRD analysis inside and outside burrows, show a clear contrast of lithology, from a muddy microcrystalline calcite matrix to a light-coloured carbonate with dolomite inside burrows (figure 9). The boundary is sharp, smooth and unlined. There is no rim cement on the burrow wall. XRD analysis also shows that quartz was detected only from the light-coloured infilling inside the burrow. The existence of quartz only inside the burrow and not the matrix suggests that clastics were transported into the open burrows after their abandonment by the trace makers.

The stable carbon isotope curve shows two negative excursions in the upper Zuun-Arts Formation (figure 1*c*). This pattern with dual negative excursions is comparable to the results from Bayan Gol and other nearby localities [10,16]. Stratigraphic successions from Morocco show similar dual negative excursions at the top of the Ediacaran [11] with slightly positive excursions below these negative spikes. The upper negative excursion in this section, in Unit 17 near the top of the Zuun-Arts, is of greater magnitude and is identifiable as the Precambrian–Cambrian boundary excursion (BACE excursion [15]) (figure 1*c*), and this negative excursion can be correlated to the BACE boundary excursion [10,16]. Based on both the first occurrence of *T. pedum* and this carbon isotope profile, the *Arenicolites* beds can be considered as located below the PC–C boundary. Even if the lower negative excursion we detected near the boundary between Units 15 and 16 marks the PC–C boundary, the lowest horizon of *Arenicolites* is still below the boundary (figure 1*b*). These lines of evidence indicate that these penetrative traces are located in the uppermost Ediacaran. Considering their co-occurrence with *Planolites*, the absolute age of these *Arenicolites* can be constrained to approximately 555–541 Ma [9].

## 5. Discussion

Modern bilaterian animals with a variety of trophic modes make U-shaped burrows in sediment as semi-permanent domiciles that are analogous to the trace fossil *Arenicolites*. *Arenicolites* is found throughout the Phanerozoic, and it frequently occurs in trace fossil assemblages that show a preference for opportunistic occupation of freshly disturbed sediment [18–21]. This also appears true of these Ediacaran *Arenicolites*, as they are preserved in deformed continental slope deposits that were presumably freshly disturbed shortly before trace maker inhabitation. While it is impossible to identify the phylum of these *Arenicolites* trace makers, they were certainly bilaterian animals based on the complexity of the traces (which cannot have been made by cnidarians or flatworms), and were likely vermiform in nature. Semi-permanent U-shaped burrows are created to bring water into one opening for feeding and respiration and to expel waste products from the other opening. This functionality only serves a trace-making animal with an anterior-posterior body axis and a complete gut: both bilaterian-grade anatomical features. Some cnidarians, such as actinians, probably made vertical burrows such as *Bergaueria*, *Conichnus* and *Dolopichnus*, but these traces are simple vertical shafts, lacking branching or U-shaped structures [22–24]. Recently, discovered putative horizontal traces from the Ediacaran of Mistaken Point are interpreted as being made by motile metazoans, probably cnidarians, not bilaterian animals [25]. Jensen *et al*. [3] stated that ‘producers other than bilaterians must be considered for simple trace fossils, and perhaps also for short-branched forms' in the Neoproterozoic. The complex U-shape burrows observed in Unit 15 of the Zuun-Arts Formation cannot have been made by cnidarians, and can safely be attributed to the activities of bilaterian animals. Therefore, the present discovery of *Arenicolites* confirms that bilaterian animals existed in the latest Ediacaran, although many studies, including molecular phylogenetics, suggests that bilaterians were present in the Ediacaran (e.g. [26]).

These penetrative trace fossils indicate that the agronomic revolution actually began in the latest Ediacaran in at least one setting. This discovery of large-sized, penetrative trace fossils is contradictory to previous studies showing that there were only small-sized penetrative traces starting in the earliest Cambrian (Fortunian) [7,8]. This finding exemplifies the sporadic and uneven development of intense bioturbation during the early evolution of animals. The agronomic revolution did not proceed in a uniform pattern of increased bioturbation levels across all depositional environments during the Cambrian radiation, but, rather, in a patchwork of varying bioturbation levels across marine seafloors that lasted well into the early Palaeozoic [27]. Palaeoenvironments with Ediacaran-style substrates and associated benthic animal adaptations are found into the middle Cambrian [28,29], and here we document a latest Ediacaran palaeoenvironment with Cambrian-style penetrative trace fossils and vertical ichnofabric development. In addition to the present discovery, there are some records of three-dimensional trace fossils recorded from the terminal Ediacaran: large trace fossils with spreiten [30], and treptichnid trace fossils [31], both from the late Ediacaran of Namibia, may also suggest the existence of bilaterian animals.

Dolomitization can also create syndepositional deformation that can sometimes form abiogenic structures resembling trace fossils [32]. However, the beds containing the vertical shafts correspond mostly to intervals consisting of non-dolomitized limestones. The vertical shafts also have consistently circular bedding plane cross-sections and possess U-shaped vertical morphologies. On the upper bed surface, these circles, as well as the longitudinal shafts, are mostly smooth in outline. These features can, therefore, be attributed to the activities of benthic animals rather than dolomitization processes. There was evidently some deformation after the formation of these burrows, altering some of the originally smooth walls into more irregular surfaces.

The maximum size of these *Arenicolites* (greater than 1 cm in tube diameter) means that the trace makers were relatively large compared to other Ediacaran, and even Fortunian, trace makers. These traces provide insight into the early evolution of bilaterians. They mark an important milestone in early animal evolution because of the behaviour they represent: they are the earliest evidence for animals making semi-permanent domiciles in sediment. The evolution of macrophagous predation was probably the selective pressure for these trace makers to build such semi-permanent infaunal structures, as they would have provided safety from epibenthic predators. Furthermore, unlike a range of typical Ediacaran horizontal trace fossils, many of which could have been formed by single-celled organisms such as protists [4], these *Arenicolites* must have been formed by trace makers that used either peristaltic motion, which requires a coelom as a hydrostatic skeleton, or excavating appendages. Non-bilaterian animals do not possess these morphological characters and can, therefore, only create simple vertical shafts. The complexity and penetrative nature of these trace fossils, therefore, provides convincing evidence for bilaterians in the latest Ediacaran just below the Precambrian–Cambrian boundary.

## 6. Conclusion

Abundant penetrative trace fossils were discovered from 11 outcrop horizons of the late Ediacaran Zuun-Arts Formation in Bayan Gol valley, Western Mongolia, as well as from float. Cross-sectional analysis of these traces shows that they penetrate at least 4 cm down from the surface, and form a U-shape. These trace fossils can, therefore, be assigned to the ichnogenus *Arenicolites*. Carbon isotope data indicate that these *Arenicolites* occur below a remarkable negative excursion assignable to the BACE event at the PC--C boundary, and below the first occurrence of *Treptichnus pedum* is at a horizon at least 130 m above these *Arenicolites*. This chemostratigraphic and biostratigraphic data shows that these trace fossils date to the late Ediacaran. Common distribution of slump folds near these trace fossils suggest its deposition on the continental slope. These abundant, large-sized, U-shape burrows show that the agronomic revolution started earlier than previously considered, and that bilaterians were present in the late Ediacaran of Western Mongolia.

## Supplementary Material

S1. Correlation of Arenicolites horizons and ratio of dolomite/calcite of the carbonate beds analyzed by XRD analysis.

## Supplementary Material

S2. Synsedimentary folding in the unit 15 near Arenicolites horizons.

## Supplementary Material

S3. Synsedimentary folding in the unit 15 near Arenicolites horizons.

## Data accessibility

Electronic supplementary material S1–S3 supporting this article have been uploaded as the Supplementary Material at *Royal Society Open Science*.
