# Supplementary figures and images for: Penetrative trace fossils from the late Ediacaran of Mongolia: early onset of the agronomic revolution
Source: R Soc Open Sci. 2018 Feb 28;5(2):172250. doi: 10.1098/rsos.172250 (PMC5830798; doi:10.1098/rsos.172250)

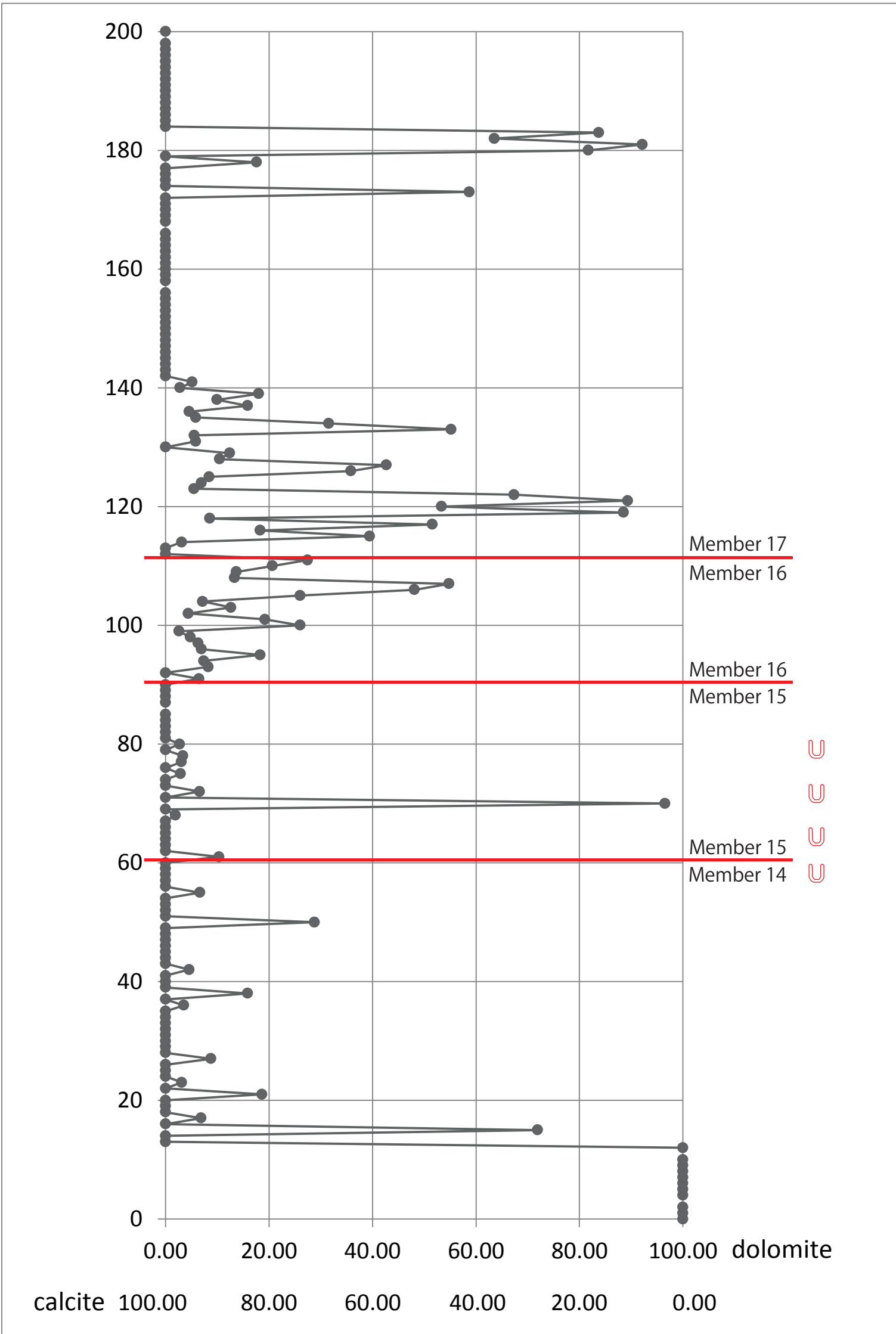

Supplement: S1. Correlation of Arenicolites horizons and ratio of dolomite/calcite of the carbonate beds analyzed by XRD analysis. [file rsos172250supp1.pdf]

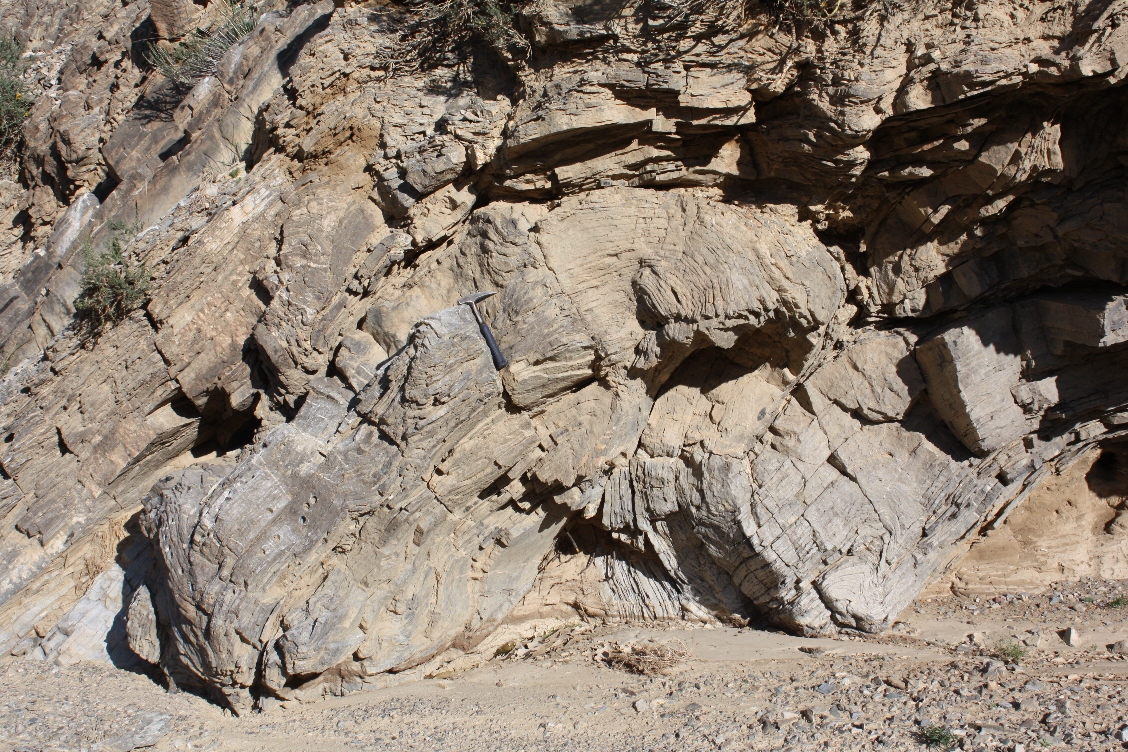

Supplement: S2. Synsedimentary folding in the unit 15 near Arenicolites horizons. [file rsos172250supp2.jpg]

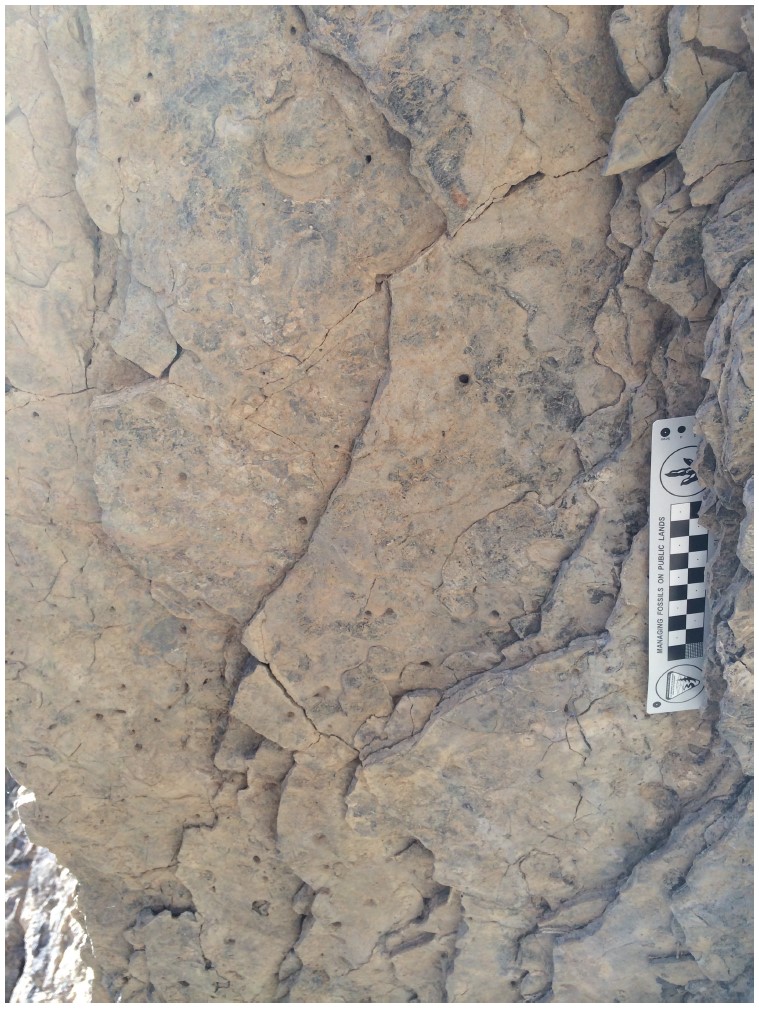

Supplement: S3. Synsedimentary folding in the unit 15 near Arenicolites horizons. [file rsos172250supp3.jpg]
